# Ultrasound assessment of muscle mass and correlation with clinical outcomes in critically ill patients: a prospective observational study

**DOI:** 10.1007/s40477-023-00823-2

**Published:** 2023-10-02

**Authors:** Cristian Arvey Guzmán-David, Héctor Andrés Ruiz-Ávila, Diana Alexandra Camargo-Rojas, Claudio Jaime Gómez-Alegría, Edgar Debray Hernández-Álvarez

**Affiliations:** 1 Master’s in Physiology, Faculty of Medicine, Universidad Nacional de Colombia, Bogotá, D.C. Colombia; 2 Intensive Care Unit, Hospital Universitario Nacional de Colombia, Bogotá, Colombia; 3 Intensive Care Research Group, Universidad Nacional de Colombia (GICI-UN), Bogotá, Colombia; 4 Kinesiotherapy Deepening Research Group, Universidad Nacional de Colombia, Bogotá, Colombia; 5 Department of Human Body Movement, Faculty of Medicine, Universidad Nacional de Colombia, Bogotá, Colombia; 6 Health and Development Research Group, Kinesiology, Universidad Nacional de Colombia, Bogotá, Colombia; 7 UNIMOL Research Group, Universidad Nacional de Colombia, Bogotá, Colombia

**Keywords:** Ultrasonography, Morbidity, Mortality, Intensive care units, Muscular atrophy

## Abstract

**Purpose:**

Muscular atrophy implies structural and functional alterations related to muscular force production and movement. This condition has been reported to be the main reason for generalized muscle weakness; it reflects the severity of the disease and can have a profound impact on short- and long-term clinical outcomes. The purpose of this study was to determine whether muscle atrophy ultrasound parameters early predict muscle weakness, morbidity, or 28-days mortality.

**Methods:**

This was a prospective, observational single center cohort study. Ultrasound was used to determine the cross-sectional area and muscle thickness of the rectus femoris on the first and third day of ICU stay. The main outcome was the incidence of significant muscle atrophy (≥ 10%).

**Results:**

Ultrasound measurements were made in 31 patients, 58% (18/31) of which showed significant muscle atrophy. The relative loss of muscle mass per day was 1.78 at 5% per day. The presence of muscle atrophy presents increased risk for limb muscle weakness and handgrip weakness. The 28-days mortality rate was similar in both subgroups.

**Conclusion:**

The presence of muscle atrophy presents an increased clinical risk for the development of limb ICUAW and handgrip, although these observations were not statistically significant. The results could be used to plan future studies on this topic.

## Introduction

Neuromyopathy due to critical illness clinically manifests muscle mass loss which reflects disease severity and affects short- and long-term clinical outcomes [1, 2]. Catabolic state and muscle atrophy are caused by various factors, inflammation, insulin resistance and hyperglycemia, muscle disuse due to rest, hormones, cytokines and pro-catabolic drugs, difficulty establishing nutritional support and advanced age. These are particularly very common in the ICU [3].

The level of muscle atrophy quantified by ultrasound has been nearly 2% per day during the first week of ICU stay [4]. In fact, it has been reported that up to approximately 45% of muscle mass such as the rectus femoris [2] can be lost after 20 days. These conditions’ structural and functional alterations in terms of muscle force and movement production, which are reported as the main reason for ICU-acquired muscle weakness (ICUAW) [5, 6] and is related to prolonged mechanical ventilation (MV), increased ICU length of stay, decreased functional status and extended mortality figurs [7].

The incidence of ICUAW is 25–31% in the medical ICU and 56–74% in the surgical ICU [8]. It is approximately 50% in patients with MV, while 70%, and even 100% in patient’s whit systemic inflammatory response syndrome and severe sepsis when the clinical situation is complicated by multi-organ dysfunction [3, 9]. Deteriorated physical, cognitive, and mental function and weakness can continue for years [10, 11] after ICU discharge, which represents a worsened quality of life, physical disability, increased healthcare costs and mortality [3].

Accurate muscle atrophy quantification as a precursor to ICUAW remains a challenge in the ICU [12–14]. Different methods directly and indirectly, quantify strength or muscle mass [15, 16] which include: functional tests, computed tomography, resonance, bioimpedance, histochemical, metabolic, biomarkers and electrophysiological. Most of the tests may have limitations referring to the patient’s difficulty in understanding and performing activities, lack of portability, radiation exposure, risk of infection, or unavailability at the patient’s bedside despite their wide variety [4, 6, 8, 17].

Therefore, sensitive and specific assessment tools available at the patient’s bedside, should be safe, preferred, cost effective, and relatively easy to use, which will allow muscle atrophy quantification and early ICUAW detection. To date, no studies have been identified in the Colombian context that uses muscle atrophy measurement parameters and their ability to predict outcomes such as muscle weakness, clinical course, and morbidity and mortality. This study aimed to define early muscle weakness, 28-days morbidity, or mortality prediction in patients with critical illnesses using muscle atrophy parameters by muscle ultrasound. The presence of muscle atrophy of > 10% in the first 72 h is believed to predict outcomes in patients in our ICU.

## Methods

### Study design and patient selection

The Research Ethics Committee of Hospital Universitario Nacional de Colombia (ID: CEI-2022-03-05) approved this prospective, observational, single-center cohort study.

Adult patients (> 18 years old), admitted to the ICU, with medical or surgical pathologies, exposed to MV for over 24 h and were expected to remain in the ICU for at least 96 h, were included. Patients referred from another institution, admitted with MV > 36 h of onset, with a neuromuscular disease diagnosis or central and/or peripheral nervous system lesions, with orthopedic limitations that hindered muscle strength assessment, or who were expected to die within the next 48 h or had decided to discontinue life support were excluded from the study.

### Cohort construction

Dynamic cohort upon ICU admission. The sociodemographic variables were recorded, and quadriceps muscle ultrasounds were mesured. Ultrasound images from two time points were analyzed: the first 24 h after admission (T1) and day 3 (T2).

Muscle strength in each patient was evaluated once they overcame their diminished state of consciousness using sedo-analgesia required for acute phase and MV management. The risk factors for muscle weakness and morbidity were recorded during the follow-up days. Morbidity including MV days, MV-free days, and ICU stay was recorded as events that occurred. Follow-up was extended until the said time was completed or earlier if the event occurred for the 28-days mortality variable. The follow-up of each patient was consequently closed in either case.

### Exposure factor definition

This cohort considered the presence of muscle atrophy, confirmed with an ultrasound of the quadriceps, as the exposure factor [9]. Methodologically, it was called an exposure factor, yet it does not correspond to intentional exposure. Muscle atrophy was considered when serial muscle ultrasound measurements reveled a change ≥ 10% [2, 9].

#### Muscle ultrasound

The ultrasound parameters used were: Rectus Femoris (RF) Cross-Sectional Area (RF-CSA) and RF Muscle Thickness (RF-MT). A 9–14 MHz linear probe connected to a Sonoscape E2 ultrasound system (Sonoscape Medical International Limited, Shenzhen, China – CMedical SAS Bogotá Colombia) was used, scanning in B-mode. A standard operating procedure was used following previously published protocols [5, 9]. Patients were positioned supine with their heads at 30°.

The measurement point was 60% of the distance between the anterior superior iliac spine and the upper edge of the patella, and the measurements were bilaterally taken [18]. The probe was placed perpendicular to the quadriceps axis, applying the lowest possible pressure, and three measurements were taken where each value (RF-CSA and RF-MT) should not exceed a 10% difference from to the previous measurements. Images were saved and tagged using ImageJ software (NIH, Bethesda, MD) [10]. After identifying the muscle tissue, the RF-MT and RF-CSA were obtained by measuring the distance or area over the inner border of the superficial and deep fascia.

### Outcome measures and data collection

The main outcome measure was the presence of muscle weakness, due to the exposure factor. Values below previously established cut-off points were recorded as a weakness: Medical Research Council Sum Score (MRC-SS) manual muscle testing (< 48 points), manual dynamometry (Jamar; Sammons Preston, Rolyan, Bolingbrook, IL) (< 7 kg in females, < 11 kg in males), and respiratory muscle strength measurements (MircoRPM Respiratory Pressure Meter, CareFusion, San Diego, USA) (maximum inspiratory and expiratory pressure of < 36 mmHg and < 40 mmHg, respectively). These measurements were performed according to previously published protocols [11, 13–16].

Secondary outcomes were: morbidity measured as the number of days on MV, ventilator-free days, and days spent in the ICU, or 28-days mortality. The following variables were recorded: age, sex, reason for ICU admission, medical history of risk factors associated with muscle weakness or an early loss of functional capacity and severity scores of the physiological alteration using the Acute Physiology and Chronic Health Evaluation II (APACHE II) and the progression of organ failure using the Sequential Organ Failure Assessment (SOFA), hemoglobin levels (g/dL), albumin (g/dL) and creatinine (mg/dL) and international normalized ratio (INR) [19]. Data reporting the risk factors associated with muscle weakness and/or atrophy were recorded [12].

### Sampling and sample size

Non-probabilistic convenience was used for sampling, where participants admitted to the ICU were prospectively recruited following the eligibility criteria. The Epi InfoTM software (version 7.2.2.16) was used to limit the sample size, and a minimum of 69 patients should be included as calculated, established under a 95% confidence level, a random error of 5%, a power level of 80%, and a ratio of unexposed/exposed of two. This document shows the preliminary outcomes, presenting results of 47% of the proposed sample.

### Statistical analysis

The IBM-Statistical Package for the Social Sciences Statistics (Version 19-Armonk, NY) and GraphPad Prism 6.0 (Boston, MA) software were used. Descriptive statistics are presented as means with standard deviation, medians with interquartile ranges, or percentages, as appropriate. The Shapiro–Wilk test was used to determine whether the variables of interest followed a normal distribution. Differences between groups were considered statistically significant when two-tailed *p* values were < 0.05. We used parametric or non-parametric tests (Student’s *t* test, *U*-Mann–Whitney, Kruskal–Wallis, Chi-square (*χ*^2^) or Fisher’s exact test) to determine the statistical differences between patients identified with or without muscle atrophy, as appropriate. The odds ratio (OR) were calculated to quantify the scope of the association between exposure variables and outcome.

## Results

### Patients and characteristics

**Fig. 1:**
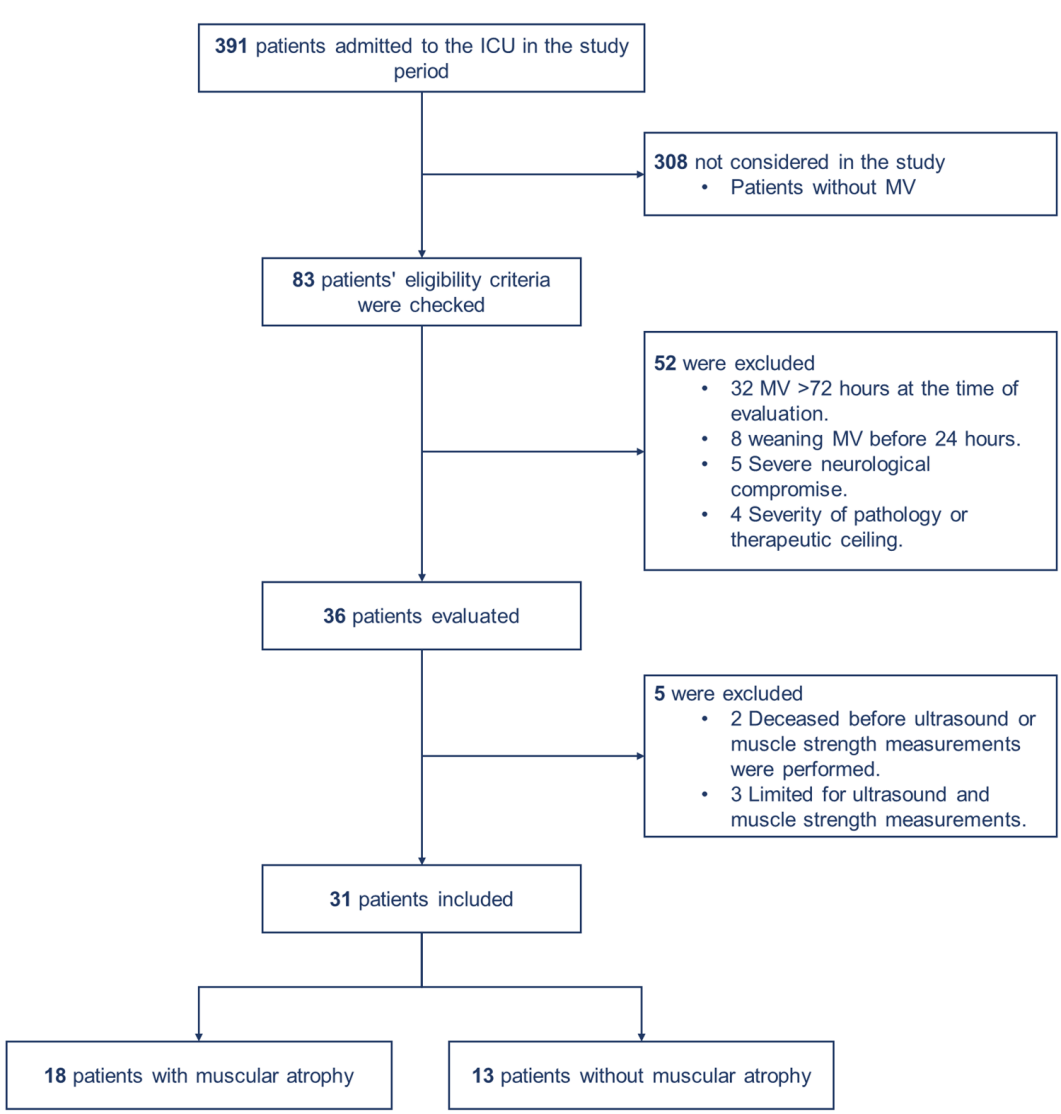
Patient eligibility flowchart; *Source*: Prepared by the author. *MV* mechanical ventilation

A total of 391 patients were admitted to a fourth-level health institution ICU from October 5 to November 30, 2022. Of these, 308 were not included in the study because they did not require MV. 83 patients were admitted or required MV (main eligibility criteria), and 52 patients were excluded (Fig. 1). The afore mentioned study period include 31 patients, of which 54.8% were male, the average age was 62.52, and the average height was 1.63 m. Further, 42% of the total cohort did not present the exposure factor, and the average age of the said subgroup of patients was 67.38. Of these, 25.8% were male. Significant muscle atrophy in the first 72 h of ICU management occurred in 58% of the total cohort in terms for patients with exposure factors. The mean age was 59 years in this subgroup of patients and distribution between genders was the same (29%) (Table 1). No significant statistical or clinical differences were reported for either subgroup in variables such as hemoglobin, albumin, and admission INR or APACHE IV. Additionally, differences in clinical terms can be considered significant in creatinine, SOFA on admission, days with MV and ventilator-free days. The main pathological history was diabetes mellitus, and the admission diagnosis was surgical.

**Table 1:** Demographic and clinical characteristics; Source: Prepared by the author

Parameters	Muscle Atrophy (*n* = 18)	No Muscle Atrophy (*n* = 13)	Total (*n* = 31)	*p*
Age (years)	59.00 ± 16.06	67.38 ± 13.51	62.52 ± 15.39	0.137
Sex (%)	29 (F) y 29 (M)	16.2(F) y 25.8 (M)	45.1 (F) y 54.8 (M)	/
Height (m)	1.64 ± 0.08	1.61 ± 0.09	1.63 ± 0.09	0.282
Weight (kg)	74.51 ± 20.49	68.46 ± 10.49	71.97 ± 17.06	0.338
Body mass index (kg/m^2^)	26.97 ± 5.71	27.95 ± 7.04	27.01 ± 5.25	0.674
Body surface area (m^2^)	1.83 ± 0.28	1.75 ± 0.16	1.80 ± 0.240	0.329
Admission Hb (g/dL)	11.08 ± 2.28	10.93 ± 1.98	11.02 ± 2.13	0.853
Admission Cre (mg/dL)	2.05 ± 1.81	1.51 ± 1.02	1.82 ± 1.53	0.342
Admission albumin (g/dL)	2.98 ± 0.58	3.28 ± 0.48	3.12 ± 0.55	0.139
Admission INR	1.13 ± 0.10	1.24 ± 0.41	1.17 ± 0.28	0.290
Admission APACHE IV	35.89 ± 15.32	33.54 ± 15.35	34.90 ± 15.12	0.677
Admission SOFA Median RIQ)	3 (2.5)	7 (6)	5 (6.5)	0.034*
MV (days)	6.00 ± 4.70	4.85 ± 3.67	5.52 ± 4.27	0.468
Days free of MV (days)	4.81 ± 3.25	6.73 ± 7.40	10.59 ± 6.57	0.367
ICU Stay (days)	10.63 ± 5.91	10.55 ± 7.75	5.59 ± 5.30	0.976
Pathological history				
None (6, 19.4%)				
Chronic obstructive pulmonary disease (7, 22.6%)				
Obesity (6, 19.4%)				
Chronic heart failure (5, 16.1%)				
Oncologic disease (6, 19.4%)				
Chronic renal insufficiency (6, 19.4%)				
Diabetes mellitus (9, 29.0%)				
ICU admission diagnoses				
Cardiovascular (13, 41.9%)				
Respiratory (11, 35.5%)				
Gastrointestinal (2, 6.4%)				
Sepsis (15, 48.4%)				
Neurological (1, 3.2%)				
Surgical (21, 67.7%)				
Metabolic (8, 25.8%)				

*Hb* hemoglobin, *Cre* creatinine, *MV* mechanical ventilation, *INR* international normalized ratio

### Risk factor behavior and its association with outcomes

**Fig. 2:**
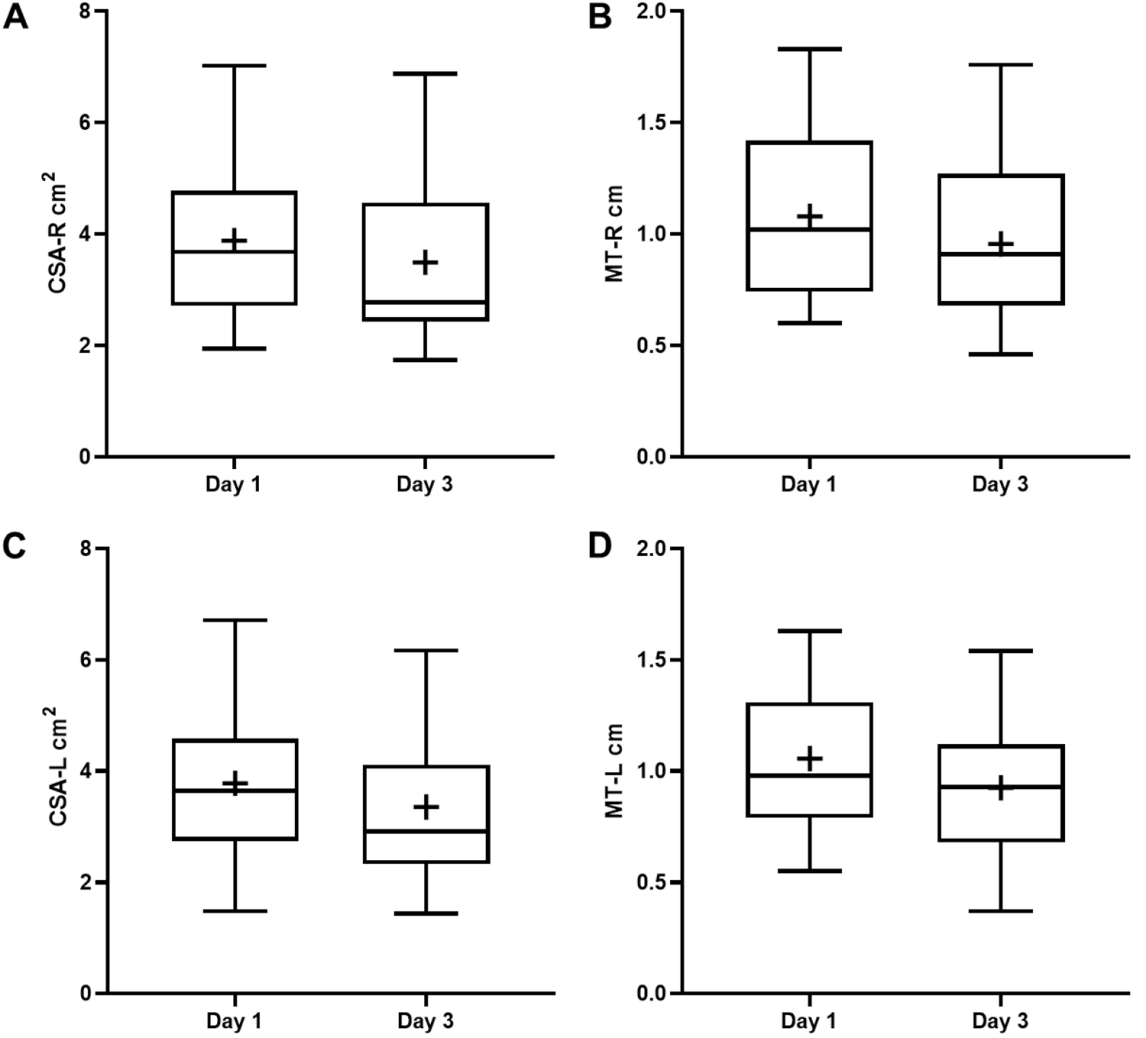
Box plot ultrasound parameters 1 and Days 3 of ICU management; *Source*: Prepared by the author in GraphPad Prism 6.0. **A** CSA-RIGHT on days 1 and 3; **B** MT-RIGHT on days 1 and 3; **C** CSA-LEFT; **D** MT-LEFT; (+) Mean Measurements performed at the baseline and 3 days later (72 h). The behavior of the ultrasound variables is reported, the distributions are wide and the average (+ sign) and median values in all the variables show a tendency to decrease when comparing both days. Right and left CSA and right MT at baseline show a symmetric distribution with similar mean and median values. However, a negative asymmetric distribution is confirmed AFTER 72 h. The distribution of the initial measurement tends to be negatively asymmetrical on MT-LEFT, and with a tendency to be positively asymmetrical at 72 h. However, a general decrease in said ultrasound parameter is confirmed when analyzing dispersion

Significant muscle mass loss (> 10%) at the ΔCSA and ΔMT parameters in 18 patients (exposed) was observed, which fluctuated from 13.02 to 16.96%. Statistically significant differences were observed in all the parameters in patients with atrophy, and the relative loss ranged from 5 to 7% (not exposed) in patients identified as conserving more muscle mass (Table 2, Fig. 2).

**Table 2:** Comparison of characteristics between patients with and without muscular atrophy; *Source*: Prepared by the author

Parameters	Muscle Atrophy (*n* = 18)	No Muscle Atrophy (*n* = 13)	*p*
Variables that determine the exposure factor			
Δ CSA-R (cm^2^)	0.525 ± 0.387	0.207 ± 0.116	0.008**
Δ CSA-L (cm^2^)	0.558 ± 0.373	0.238 ± 0.187	0.008**
Δ MT-R (cm)	0.172 ± 0.097	0.056 ± 0.051	0.001**
Δ MT-L (cm)	0.186 ± 0.108	0.055 ± 0.029	0.000**
Percentage loss CSA-R (%)	13.02 ± 8.18	6.43 ± 3.35	0.011*
Percentage loss CSA-L (%)	13.31 ± 8.04	7.25 ± 5.28	0.025*
Percent MT-R loss (%)	14.73 ± 6.37	5.39 ± 3.58	0.000**
Percent MT-L loss (%)	16.96 ± 7.99	5.73 ± 2.97	0.000**
Muscle strength outcomes			
MRC	39.28 ± 15.04	40.23 ± 19.02	0.877
Dynamometry R (kg-F)	12.17 ± 9.22	16.85 ± 9.97	0.188
Dynamometry L (kg-F)	12.28 ± 9.50	17.69 ± 9.93	0.135
MIP (cmH_2_O)	28.83 ± 15.09	27.77 ± 15.03	0.848
MEP (cmH_2_O)	36.39 ± 20.80	38.31 ± 21.52	0.805
Morbidity outcomes			
MV (days)	6.00 ± 4.70	4.85 ± 3.67	0.468
MV-free days (days)	4.81 ± 3.25	6.73 ± 7.40	0.367
ICU stay (days)	10.63 ± 5.91	10.55 ± 7.75	0.976
Mortality			
Mortality at 28 days (%)	16.6	23	0.669
Risk factors			
Admission APACHE IV	35.89 ± 15.32	33.54 ± 15.35	0.677
Admission SOFA	7 (6)	3(2.5)	0.034*
Analgesia (days)	9.56 ± 4.85	10.00 ± 7.09	0.837
Sedation (days)	5.44 ± 4.72	4.23 ± 4.02	0.459
Noradrenaline (days)	4.17 ± 2.48	3.92 ± 4.46	0.847
Vasopressin (days)	0.78 ± 1.22	0.85 ± 2.23	0.913
Dobutamine (days)	1.33 ± 1.97	2.23 ± 4.11	0.424
Antibiotic (days)	4.72 ± 6.27	3.00 ± 5.00	0.419
Statins (days)	3.22 ± 6.255	7.77 ± 8.671	0.100
Neuromuscular blockade (days)	0.06 ± 0.236	0.31 ± 1.109	0.354
Corticotherapy (days)	1.28 ± 1.179	1.31 ± 1.843	0.956
Hyperglycemia (events)	14.83 ± 10.562	15.31 ± 9.313	0.898
Transfusion Support Red Blood Cells (%)	55.56	61.54	0.749
Transfusion Support Platelets (%)	16.67	15.38	0.927
Transfusion Support Plasma (%)	11.11	15.38	0.737
Oral Nutrition (%)	55.56	69.23	0.457
Enteral Nutrition (%)	72.22	76.92	0.777
Parenteral Nutrition (%)	27.78	23.08	0.777

*MIP* Maximum Inspiratory Pressure, *MEP* Maximum Expiratory Pressure

**Significance level < 0.01

*Significance level < 0.05

**Fig. 3:**
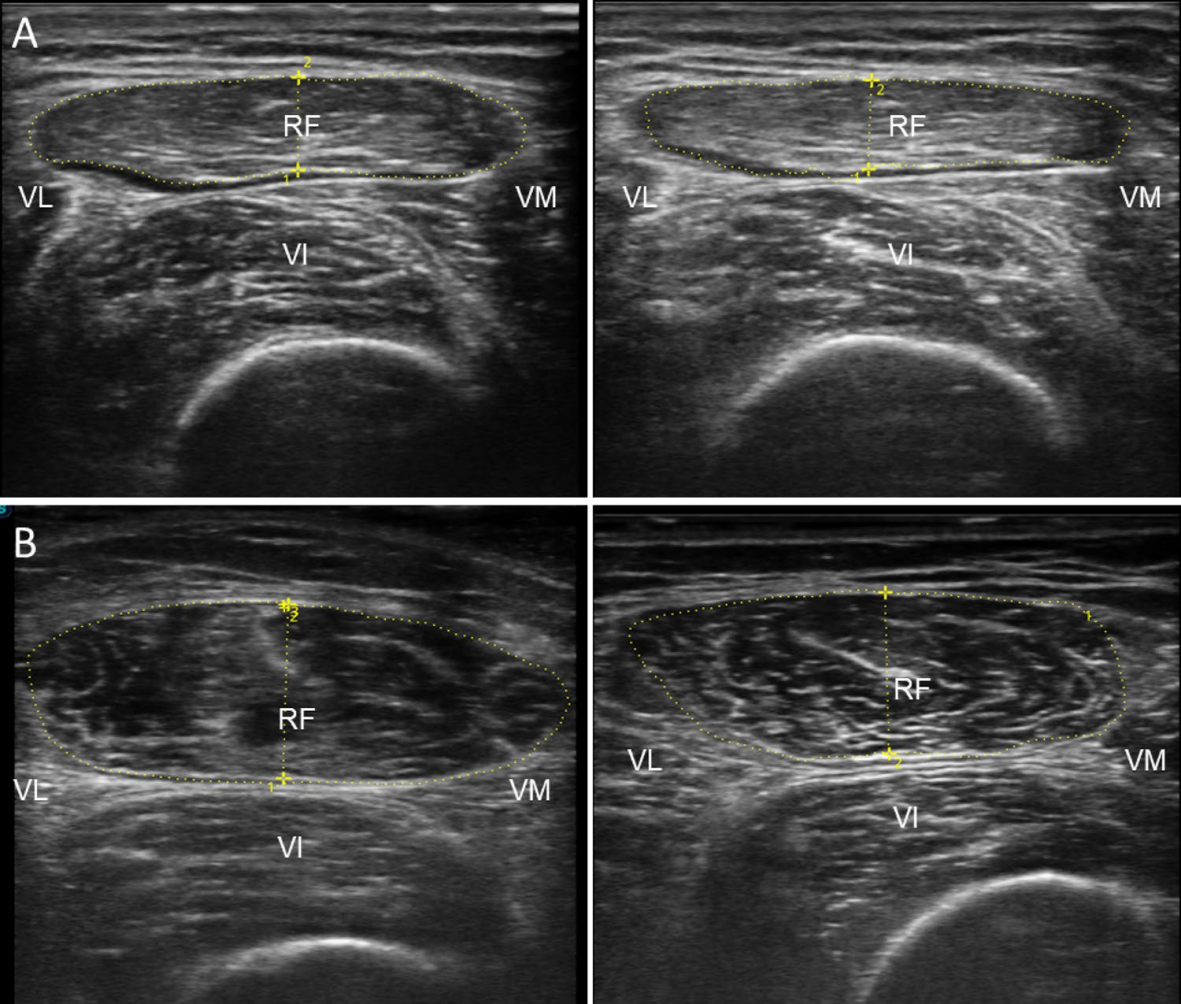
Muscle Ultrasound of Patients at Baseline and after 72 h. **A** and **B** Indicate ultrasounds of different patients, left panel ultrasounds at the baseline and right panel after 72 h—*RF* Rectus Femoris, *VI* Vastus Intermedius, *VL* Vastus Lateralis, *VM* Vastus Medialis

Figure 3 shows ultrasound scans of patients at the baseline and after 72 h, qualitatively presenting a decrease in the cross-sectional area, muscle thickness and increased space at the intra and interfascial level, and changes in the level of echogenicity that could be associated with the change in muscle quality (although this was not analyzed in this study).

The average values of limb muscle strength with MRC and respiratory muscle strength were similar in both subgroups but manual dynamometry values were lower in patients with muscle atrophy. Among exposed patients, 38.7% presented an ICUAW outcome evaluated with MRC, 19.3% with dynamometry and 38.7% with respiratory muscle strength tests. No statistically significant differences were observed, but, a difference in clinical terms in the dynamometry scores between the subgroups can be considered relevant (Table 2).

The total days of MV were higher in patients with the exposure factor, although the mean difference was approximately 1.2 days. ICU stay was similar in both subgroups. This indicated a greater number of IMV-free days in the non-exposed subgroup but was not statistically significant. Mortality at 28 days occurred in six patients (19.3% of the total cohort), and distribution was the same in both subgroups (Table 2).

The total number of days that patients received analgesia, sedation, hemodynamic support, antibiotics, statins, neuromuscular blockade, corticosteroids, or the average number of hyperglycemic events occurring during the ICU stay revealed no differences, as well as the proportion of patients who received transfusion support or according to the type of nutritional support (Table 2).

The presence of muscle atrophy, which was considered the exposure factor in this cohort, was contrasted with different outcome variables to determine the degree of association. The presence of muscle atrophy represents a risk of 3.2 (OR 3.2 95% CI 0.724–14.145; *p* > 0.05) and 2.75 (OR 2.75 95% CI 0.456–16.592; *p* > 0.05) times greater for presenting limb muscle weakness and for hand grip respectively. However, these findings were not significant. Conversely, respiratory muscle weakness, MV of > 7 days or, a mortality outcome revealed no association, because these outcomes occurred similarly in both subgroups (atrophy, and no atrophy).

## Discussion

This study aimed to determine early muscle weakness, morbidity, or 28-days mortality prediction in patients in the ICU using ultrasound parameters of muscle atrophy. To our best knowledge, no study has been conducted in Colombia for this purpose. The main findings in this study are:

- Consistently decreased muscle mass in patients with critical illnesses in a relatively short period (72 h) and the presence of muscle atrophy in the entire cohort. However, muscle atrophy measured with CSA and MT muscle ultrasound parameters exceeded the 10% threshold in 58% of all patients.
- A value ≥ 10% muscle mass loss by ultrasound (CSA or MT) in the first 72 h of ICU management could increase the probability of developing ICUAW. In fact, the presence of muscle atrophy represents a risk 3.2 times greater than ICUAW and 2.75 times greater for handgrip weakness.
- The ultrasound measures of muscle atrophy analyzed in this cohort did not show significant associations with the main outcome measures. This is probably because the similar characteristics of patients considered with or without muscular atrophy.
- To our best knowledge, no ICU study has used a ≥ 10% reduction in CSA or MT as a cut-off point to indicate significant muscle atrophy in the first 72 h. This study revealed a similar incidence of early significant muscle atrophy of 58%, to previous report by Bloch et al. [21, 22] and Hrdy et al. [20], this incidence was identified with an accelerated muscle mass loss in the first 72 h in the ICU in this cohort.

The muscle atrophy observed by MT and/or CSA of the rectus femoris was approximately 1.79–5% daily. This is worrisome in our context, since studies recently compiled in the systematic review and meta-analysis conducted by Fazzini et al. [23] revealed that the muscle mass loss daily can be approximately 2%. No particular factors explain this finding, beyond a higher average age and patients with two or more comorbidities, although the CSA and MT values at the baseline of the follow-up in this study were similar to those reported in healthy people, even in studies conducted in places different from ours [24, 25].

This study revealed muscle atrophy despite usual rehabilitation. Decreased contractility and muscle mass loss can independently occur, therefore, muscle atrophy in ICU and ICUAW should not be interpreted as synonymous or interchangeable [26], although the former may cause the latter. The physiological and clinical studies of the muscle mass loss and myopathy that explain ICUAW development have described four possible causal events that may indicate its onset although they cannot be specifically detailed with ultrasound [27].

Our findings on muscle mass reduction partially coincided with what has been described in other studies in critically ill populations, although little research has been conducted to assess the muscle mass loss at 72 h. Parry et al. [5] conducted serial measurements at admission, 3, 7, and 10 days in 22 patients with critical illnesses receiving MV. The percentage of average muscle mass loss in the CSA and MT variables ranged from 0.2 to 9% in the first 72 h, while the reduction in muscle mass was from 5.39 to 16.96% in this study. However, age and APACHE scores were lower than what was observed in our cohort, 56 ± 18 years and 23 ± 8 years, respectively.

This last observation is relevant because age and pathology severity have been considered determinants of the muscle mass loss, among the risk factors for presenting myopathy in the ICU [28].

The previously reported extent of muscle mass loss may be within the values reported for later follow-ups. A decrease in MT from 5.9 to 24.9% and CSA from 12.1 to 23.2% have been reported compared to the baseline value after analyzing muscle mass reduction on day 7 [29, 30]. This accounts for a large proportion of early muscle atrophy. However, muscle wasting is best assessed at 7 days, because this day could represent accumulated muscle wasting [31]. Thus, special attention can be beneficial for patients with muscle loss in addition to a more aggressive therapy aimed at mitigating this problem through nutritional and rehabilitation strategies [23, 31].

The last observation was different because the extent of muscle atrophy was considerably high in the first 72 h in our cohort, and the first hours of management of a patient with a critical illness seem crucial [32]. The muscle mass loss can be mitigated with strategies implemented the moment the patients enter the ICU [33].

Hence, the clinical and paraclinical variables demonstrated improvement in the first 72 h, and these observations were significant in clinical and statistical terms. However, muscle atrophy was high, and the muscle mass loss continued as observed in other publications, despite overcoming the most acute phase of a critical illness [23, 34].

A systematic review and meta-analysis by Fazzini et al. [23] reported up to 24.5–29.4% muscle mass loss measured with MT and CSA respectively, at 14 days of follow-up in the ICU. Up to 45% of the muscle mass of the rectus femoris can be lost after 20 days [23] a greater catabolic state where the release of hormones such as norepinephrine, cortisol, and glucagon increase gluconeogenesis, glycogenolysis, free fatty acid mobilization, and muscle proteolysis, explains this muscle mass loss in the initial or acute phase. This phase occurs in critical illness as a strategy to increase energy availability [35, 36]. Consequently, effector anabolic hormones decrease, which may explain muscle atrophy that could contribute to “myogenic origin” weakness together with immobilization [37].

Patients with organ failure lost muscle mass early and that the loss was more severe compared to patients with less involvement (lower severity scores) [38]. The CSA changes of the rectus femoris were significantly higher In patients with sepsis and septic shock, especially in patients with MV [30]. Our study confirmed this result, and the proportion of patients with sepsis or septic shock represented 35.4% and all presented muscle atrophy.

On the other hand, this cohort included elderly patients with pathological history such as chronic respiratory, cardiovascular and renal disease and oncological pathologies, all of which seem to induce muscle atrophy through a common pathway that is the activation of the renin angiotensin aldosterone system (RAS), An active RAS with elevated angiotensin II levels is harmful, producing: insulin resistance, muscle atrophy and fibrosis. It has recently been reported that angiotensin-converting enzyme (ACE) inhibitor drugs appear to have preventive effects against the development of muscle atrophy. This could be studied as a pharmacological management strategy along with early mobilization and exercise strategies to “prevent” loss of muscle mass in the first 72 h [39, 40].

Muscle atrophy was not significantly correlated with outcomes such as limb ICUAW, handgrip weakness, respiratory muscle weakness, prolonged MV, or ICU stay. However, ICUAW occurred in 54.8% of the cohort, handgrip weakness in 25.8%, respiratory muscle weakness in 70.9%, and prolonged MV (> 7 days) in 22.5%. These findings are consistent with other studies, where the ICUAW ranges from 25 to 31% in the medical ICU and from 56 to 74% in the surgical ICU. This work was developed in a polyvalent ICU where the proportion of enrolled surgical patients was close to 70% [41, 42].

The presence of muscle atrophy proved to be a clinically considerable risk for the presence of muscle weakness measured with MRC and with dynamometry, with a risk of 3.20 and 2.75 times higher. Moreover, some patients have a lower extent although muscle atrophy was present in the entire cohort, and it was observed early (first 72 h). This is because a 5 or 7-days follow-up will reveal a greater muscle mass loss, and it will be associated more with the outcomes registered in this cohort, as described in other studies [31, 43]. The degree of muscle mass loss was not associated with prolonged MV, ICU stay, or mortality in this cohort.

In contrast, studies such as the one by Nakanishi et al. [44], did not demonstrate that muscle atrophy measured with MT and CSA of the quadriceps and biceps brachii could predict in-hospital mortality when said measure of muscle alteration was determined on the third day of ICU management. However, the observations of biceps brachii atrophy on days 5 and 7 demonstrated a greater association with in-hospital mortality. For their part, Dusseaux et al. [43], in a retrospective study analyzing 25 patients, did not the association of alterations in paravertebral muscles with mortality.

## Limitations

Several limitations should be emphasized when interpreting the findings of this study:

- This is a single-center study with a small sample size, thus power, precision, and external validity are limited.
- The result may not be generalizable to other muscle groups, since only the quadriceps muscle was evaluated. However, the quadriceps is the most studied muscle in the ICU population, considering that it is an important antigravity, weight-bearing muscle and is associated with physical function outcomes [26]. It is also easily accessible in the supine position and has been reported to have excellent association with whole-body muscle mass [45].
- We only measured RF-CSA and RF-MT, however, the adjustment of variables such as quadriceps thickness (rectus femoris and vastus intermedius) with measures such as BMI (STAR—Sonographic Thigh Adjustment Ratio) can provide information on the baseline condition (without requiring follow-up measures) have shown a correlation with functional tests such as Timed UP and Go, Chair Stand Test, gait speed, muscle strength and sarcopenia [46, 47].
- No nutritional data was available beyond determining the type of nutrition they received during their ICU stay, thus the association between nutritional support and muscle status cannot be determined.
- Biomarker measures that may indicate the physiological/pathophysiological state of muscle atrophy at the systemic level were not included.
- The presence of many confounding factors such as age, sex, disease severity, comorbidities, and risk factors themselves, can affect both muscle thickness and the results. However, we were unable to perform optimal statistical analysis to adjust them due to the limited number of events.
- The inclusion of other ultrasound-derived measurements, such as echogenicity and pennation angle, may provide additional information on muscle health in critical illness. However, this was outside the scope of this study, which is a fact that does not necessarily invalidate the presented findings.
- Lastly, the impact of muscle mass loss with measures related to physical capacity and/or functionality in the ICU or at discharge (IMS, PERME, PFIT-s, CPAx, 6-min walk) or recovery from long term were not analyzes.

## Conclusions

Patients with critical illnesses present a consistent muscle mass loss in a relatively early period (1.78–5% daily), which is substantially higher than that recently reported. The presence of muscle atrophy presents an increased clinical risk for developing limb ICUAW and handgrip, although these observations were not statistically significant.

Muscle ultrasound in the ICU is a very useful tool for early detection and monitoring of muscle mass loss, it provides tools to determine individualized interventions such as rehabilitation and nutrition, and it has prognostic implications. Therefore, clinical studies are necessary to improve understanding of the mechanisms underlying muscle atrophy in critical illnesses and to inform the development or modification of interventions for its prevention or mitigation.

## Data Availability

The data that support the fndings of this study are available from Hospital Universitario Nacional repository (http://aplicaciones.hun.edu.co/redcap/). Restrictions apply to the availability of these data, which were used under license for this study. Data are available with the permission of Hospital Universitario Nacional, contact information: investigacion@hun.edu.co.
